# Intra-Group Lethal Gang Aggression in Domestic Pigs *(Sus scrofa domesticus)*

**DOI:** 10.3390/ani10081287

**Published:** 2020-07-28

**Authors:** Irene Camerlink, Jen-Yun Chou, Simon P. Turner

**Affiliations:** 1 Institute of Genetics and Animal Biotechnology, Polish Academy of Sciences, Ul. Postępu 36A, Jastrzębiec, 05-552 Magdalenka, Poland; 2 Animal Behaviour & Welfare, Department of Animal and Veterinary Sciences, Scotland’s Rural College (SRUC), West Mains Rd., Edinburgh EH9 3JG, UK; jenyun.chou@gmail.com (J.-Y.C.); simon.turner@sruc.ac.uk (S.P.T.); 3 Institute of Animal Welfare Science, Department of Farm Animals and Veterinary Public Health, University for Veterinary Medicine Vienna, Veterinärplatz 1, 1210 Vienna, Austria; 4 Pig Development Department, Teagasc, P61 P302 Moorepark, Ireland; 5 Royal (Dick) School of Veterinary Studies, University of Edinburgh, Easter Bush, Midlothian EH25 9RG, UK

**Keywords:** aggression, farm, death, *Sus scrofa*, social behaviour, animal welfare, victimization, coalition, attacks

## Abstract

**Simple Summary:**

Aggression between pigs in pig husbandry is common during regrouping but rare in stable social groups. Farmers report the occurrence of lethal gang aggression in stable groups of pigs, whereby the group attacks one group member until it is dead. Our aim was to document this extreme type of aggression and to identify potential causes. Forty-two farmers, experiencing lethal gang aggression or not, filled out a survey about their farm management. From 91 victims, information was obtained on their injuries and body condition. Gang aggression occurred more on farms with deep straw bedding, a housing type commonly associated with better animal welfare. However, the presence of straw may also be related to other factors, which could not be disentangled here. Gang aggression did not relate to the genetic line, breeding company or feed type. It equally occurred between females and males and tended to occur more in winter. Victims were covered in injuries, but had a healthy body condition, whereas survivors had a lower body condition. Overall, the cause seems multi-factorial, and further research on the occurrence of lethal gang aggression is needed.

**Abstract:**

Intraspecific coalitional aggression is rare among all species, especially within stable social groups. We report here numerous cases of intraspecific lethal gang aggression within stable groups of domestic pigs. The objective was to describe this extreme aggression and to identify potential causes. Management data were collected from farms with (*n* = 23) and without (*n* = 19) gang aggression. From one farm, 91 victims were assessed for skin injuries and body condition score. Lethal gang aggression was significantly associated with deep straw bedding, which may be related to various other factors. Gang aggression tended to occur more in winter, and was unrelated to genetic line, breeding company, group size or feed type. It occurred equally in female-only and mixed sex groups (male-only groups were not represented), from around eight weeks of age. Injuries typically covered the whole body and were more severe on the front of the body. Victims who survived had a lower body condition score and fewer injuries than victims found dead. There are still many unknowns as to why this abnormal social behaviour occurs and it deserves further research attention, both for its applied relevance to animal welfare as for the evolutionary background of lethal gang aggression.

## 1. Introduction

Aggressive behaviour has a clear evolutionary benefit as it may provide the aggressor access to resources necessary for survival [1,2]. However, this has to be balanced against the costs of fighting, and its potential risks for survival [3,4]. Sherrow [5] separates aggression into lethal or non-lethal based on the outcome to the victims. Newton-Fisher & Thompson [2] further divide lethal aggression into infanticide, dyadic, and coalitional aggression. A coalition is defined as individuals in a social group supporting each other during agonistic encounters [5]. Most cases of intraspecific coalitional aggression are recorded in primates [6], where reports of chimpanzee warfare have drawn much attention [7,8]. A group of male animals may attack in a coordinated manner and kill members from a neighbouring group, either to gain territory, food resources or females [9]. Within-group lethal coalitional aggression is rare, but has been observed when a group of adult subordinates jointly challenge the alpha male [10,11], or when a sudden drop in the fertile female-to-male ratio occurs [10]. These occurrences often relate to a shift in dominance relationships, but the reasons for the lethal outcomes are in most cases unclear.

In recent years, farmers have notified farm advisors and researchers that domestic pigs may suddenly jointly attack and kill a known group member that they have cohabited with for a prolonged period. The number of reported cases of this coalitional lethal aggression in pigs is much higher than any reports from other species. Moreover, coalitional aggression in the wild often involves only a few individuals who form a coalition [12,13], whereas here, according to the reports from practice, a group turns towards one individual; hence the term ‘gang aggression’ used in this context. We define intra-group lethal gang aggression as a group of animals that jointly attacks one of its own familiar group members until it is killed. This type of aggression is said to have occurred occasionally since the 1950s, but has not been documented in domestic pigs or wild boars. Meese and Ewbank [14] did mention that sudden outbreaks of violent fighting with the killing of group members were not uncommon in commercially raised pigs. The behaviour is in sharp contrast to the normal behavioural repertoire. 

The domestication of the wild boar (*Sus scrofa*) into the domestic pig (*Sus scrofa domesticus*) has hardly altered the natural behavioural repertoire and the motivation to establish and maintain dominance relationships [15]. Fights between adult wild boar males during the breeding season can cause fatal injuries [16], but conflicts, in wild boar and domestic pigs, are mainly solved through agonistic displays, such as threats and withdrawal [17,18]. During the formation of new social groups, competition for rank can be intense and typically results in skin lesions, but between pre-pubertal animals rarely results in death. The level of aggression during regrouping in commercial pig husbandry is starkly higher than in nature, and has been extensively studied as an animal welfare issue [19]. In familiar groups of pigs, when dominance relationships are settled, aggression is minimal [20]. 

The emergence of intra-group lethal gang (coalitional) aggression in domestic pigs represents a major departure from the behavioural repertoire of their ancestors and its cause is unknown. As intra-group lethal gang aggression in pigs has never before been scientifically reported or investigated, the aim of this study was to describe its occurrence and to make a first step to investigate its possible causes.

## 2. Materials and Methods 

### 2.1. Ethical Statement

This study did not include an animal experiment and therefore did not require ethical approval from an animal experimental committee. Approval of the Human Ethics Research Committee was not required. The photos of victims can be shocking and by no means reflect the average farm situation. These images are shown to reflect the average severity of this specific lethal gang aggression only. 

### 2.2. Farm Information

Farm data were collected through a survey by purposive sampling focusing on aggression in static groups of pigs, i.e., in groups that have not changed group composition recently. The survey was distributed initially by post (leading to four responses) and by e-mail to farmers and farm advisers who contacted us about this problem (leading to two responses). From December 2017, the survey was placed online using SurveyMonkey, and the link was distributed to farmers and farm advisors through email, farmers’ meetings in the UK, and through an international farmers’ magazine (37 responses). In total, 43 responses were received. Responses originated from the UK, USA, Canada, Australia, The Netherlands, and Germany. Respondents had good command of the English language. One response was discarded due to a mostly incomplete answer. Firstly, respondents were asked in an open question to describe the situation at their farm regarding aggression between pigs in static groups. Then they were asked, in four multiple-choice and five open questions, to provide information on the frequency of the aggression occurring, whether the groups consisted of males or females, the age of the animals, the consequences to the victim, the genetics, housing conditions, and nutrition. The survey is available in Appendix A Table A1. Several respondents also sought contact by e-mail and further details provided by e-mail were added as additional information to their survey response. Text was labelled based on keywords, such as ‘gang’ and ‘death’ and appropriate synonyms to categorize the information. The farms were categorized into either having lethal gang aggression (*n* = 23) or not (*n* = 19). The distinction was made based on the respondents’ description of the aggression matching the definition of lethal gang aggression (e.g., including the words gang, death, kill, or team-up in their open answers) or if they indicated that the victim died as a result of aggression. The breed or genetic line was categorized into purebred (damline or purebred victims), cross bred (commercial crosses of white type pigs), and Duroc cross bred (crossbreed between any white type dam and a Duroc sire). 

### 2.3. Victim Information

Data on 316 deaths due to this type of aggression were collected on a focal farm over the course of two years. The farrow-to-finish farm, located in the UK, had issues with lethal aggression for several years and had kept a precise record of all attacks, including images of all victims in 2016. The growing pigs (commercial crossbreeds) were kept as mixed sex groups (gilts and boars) on deep straw bedding in large groups of 100. Space allowance, feeder space, and drinkers met the legal requirements. 

For 209 victims (recorded from 2014–2016), the date of attack, the number of days the animal was in the group since entry, and the estimated body weight were available. Estimated body weight in kg (BWe) was determined for growers (4–12 weeks of age) as BWe = 7 kg + 0.6 * N Days, and for finishers (12–22 weeks of age) as BWe = 40 kg + 0.9 * N Days, where “N Days” is the number of days that the animal was in the group. For 16 pigs, only the date of death was recorded. These data were used to assess seasonal influences and weight distribution. 

**Figure 1:**
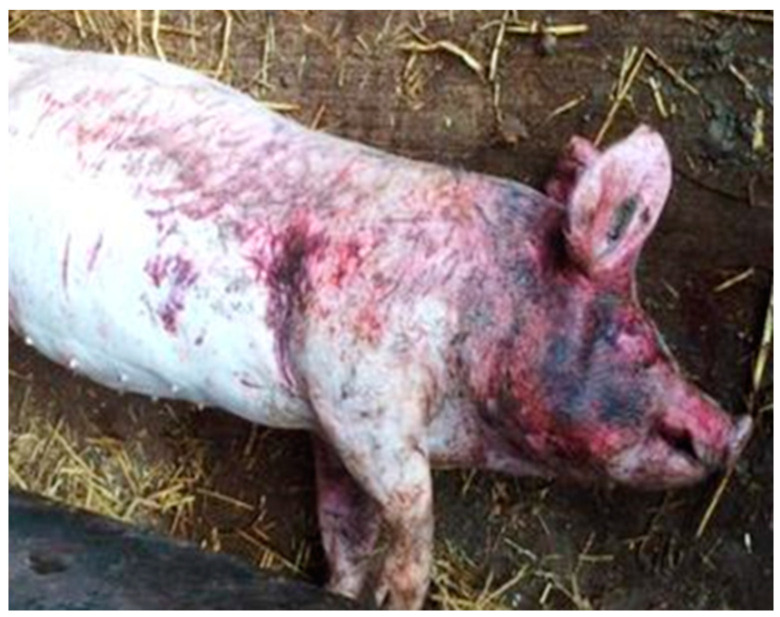
Extreme patches of skin lesions on the body, predominantly in the face, of a dead victim.

Another 91 victims, recorded in 2016, had been photographed when they had been removed from the pen by farm staff. The photos were used for assessing the body condition of the animal (score 1: emaciated; 2: thin; 3: ideal; 4: fat; 5: obese) and the number of skin lesions (score 1–5; Table 1). Scores were given separately for the face (face and ears, up to the cheek), shoulder (front region from the neck to the front leg), middle (back and belly), and rear (hind quarters and hind leg) of the body. The classification of scores and the selection of the body regions were adapted to the severity of gang aggression. For example, pigs had patches of large numbers of lesions (Figure 1), which is not commonly observed when scoring skin lesions, and falls outside of existing scoring methods. Data on the skin lesion score and body condition score were used to assess the type and severity of aggression and the clinical health of the victim. From the 91 photos of victims, it was also recorded whether they were alive (survived) or not. In total, 20 out of the 91 pigs were alive when they were removed from the pen.

### 2.4. Data Analysis

Data were analysed in SAS version 9.3 (Copyright ^©^ 2016 SAS Institute Inc., Cary, NC, USA). Data are presented as percentages and means with standard error (SEM) unless stated otherwise. *p*-Values of <0.05 were considered significant whereas values >0.05 but <0.10 were reported as tendencies. Odds ratio estimates were reported with their 95% Wald confidence interval (CI).

#### 2.4.1. Analyses of Farm Survey Data (*n* = 42) 

Multiple choice answers were expressed as a percentage of all responses for that question. Open questions were classified by reoccurring words (e.g., health, gilt, gang). Descriptive statistics (frequencies and means with standard deviation and range) were obtained over the number of responses per question. The potential causes of lethal aggression were analysed over all farm survey data, using as a response variable whether farms experienced lethal gang aggression or not. Using logistic regression, the response variable ‘lethal aggression’ (yes/no) was analysed with the following fixed effects: group composition (females only, males only, or mixed sex groups), age category (growers, 4–12 weeks of age; finishers, 12–22 weeks of age; breeding animals, >22 weeks of age; other, all other age groups), group size (<30, 31–100, >100 or variable across the farm), housing type (indoor unbedded, deep straw, or other), breeding company (7 categories), breed (purebred/crossbred/Duroc crossbred), feed type (dry, wet or mixed), and feed source (feed company or home milled). Each fixed effect was analysed separately, as a main effect, for its association with the occurrence of gang aggression, as well relevant interactions, for step-wise addition to the model. However, only the fixed effect “housing type” had a *p*-Value < 0.10 and was retained. Results of the logistic regression model are presented as odds ratios (OR) with their confidence interval (CI). 

#### 2.4.2. Analyses of Victim Records (*n* = 316)

The total number of attacks per month per year due to lethal aggression was analysed in a mixed model (MIXED) with season as a fixed effect and year as random effect to account for non-independence of months within a year. The residuals of the model were normally distributed (Shapiro-Wilk 0.94). Results of the linear model are presented as means ± SEM.

#### 2.4.3. Analyses of Victim Data Obtained from Images (*n* = 91)

The likelihood of survival (survived yes/no) was analysed using logistic regression with the pigs’ body condition score and average skin lesion score, or the score per body part, as fixed effects. Results are presented as means ± SD and OR with CI. The skin lesion score was analysed as the response variable in a generalized mixed model (GLIMMIX) using a Poisson distribution with a log link function, with the location of the lesions (face, shoulder, middle or rear) as a fixed effect and the individual as a repeated effect to account for multiple observations per animal. The results are presented as means ± SEM.

## 3. Results

### 3.1. Incidence of Gang Aggression on Affected Farms

**Figure 2:**
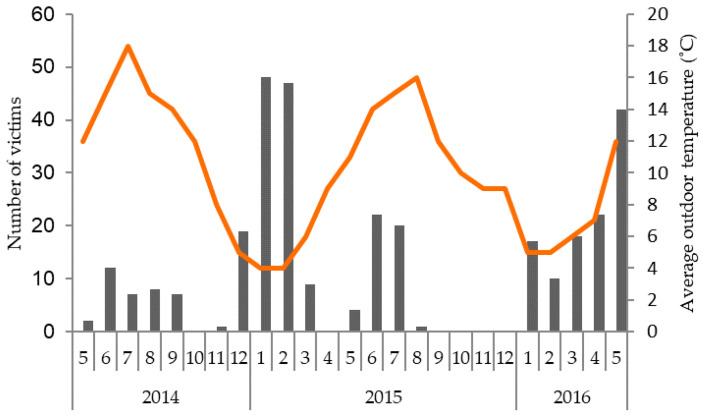
Number of cases of lethal gang aggression (*n* = 316) from 2014–2016 (months on the *x*-axis) at the focal farm for which accurate mortality data were available. This farm reported 2.6% mortality due to gang aggression. The secondary axis shows the average monthly outdoor temperature (in degrees Celsius) for the farm location.

Lethal gang aggression was reported across countries, including the UK, Canada, North America, Germany and The Netherlands, showing no geographical trend. Farmers on affected farms (*n* = 23) reported that it occurred in phases (34.8%), 1–2 times per month (26.1%), occasionally (26.1%), or frequently (13%). Data from the focal farm, including 316 attacks, showed a trend of seasonal influences (F_3,19_ = 2.86; *p* = 0.06; Figure 2), with significantly more cases in winter (23.5 ± 5.38) as compared to autumn (1.3 ± 5.38) (Post-hoc comparison: *p* = 0.009). The number of cases in spring (13.9 ± 4.98) and summer (11.7 ± 5.38) did not significantly differ from those of the other seasons. 

Lethal gang aggression within static groups of pigs was found to cause a 6% mortality rate on one farm, and others reported 1.0–2.6% mortality in their open answers. The duration from the start of the attack until the victim died, as reported by three respondents in the survey, ranged from half an hour to 24 h. Quotes from the respondents related to the duration and other characteristics of lethal gang aggression are provided in Appendix B Table A2.

### 3.2. Potential Causes

A comparison between farms that experienced lethal gang aggression against farms revealed that the only external factor significantly associated with lethal gang aggression was the use of deep straw bedding (χ = 8.467, *p* = 0.01; Table 2). For pigs housed on straw bedding, the odds ratio of lethal gang aggression was 28.16 times higher than that for conventionally housed pigs (indoor barren pens) (straw vs. barren: 28.16, CI: 2.962, 267.780). The odds ratio for gang aggression between pigs with outdoor access or being kept fully outdoors, as compared to indoor barren housing, was 2.167 (CI: 0.334, 14.057). 

Group size was mostly less than 30 animals, especially for farms without gang aggression (Table 2), or 30–100 animals. Group size varied for indoor barren housing and straw bedded systems, with no farm with large groups (>100) in barren housing conditions but three farms with large groups on straw. As numbers per category were low, there was no significant interaction between group size and housing condition for the occurrence of gang aggression (*p* > 0.10). Group composition did not significantly differ between farms with or without lethal gang aggression, despite a much higher percentage of female-only groups on farms with lethal aggression (Table 2). 

The pig breeds included various combinations of Large White, Landrace and Duroc crossbreeds and synthetic lines as well as damlines, to rare breed pigs. The occurrence of purebred, crossbred and Duroc crossbred pigs did not significantly differ between farms with or without gang aggression (Table 2). The genetics originated from at least six different breeding companies (seventh category includes ‘non-disclosed’). The farms with lethal gang aggression did not significantly differ in their choice of breeding company as compared to farms without lethal aggression (Table 2) nor in their feeding strategy (wet, dry or mixed) and feed supply (feed company vs. home milling; Table 2). 

### 3.3. The Victim

Dates of attacks confirmed that the aggression took place in static groups. Victims from the focal farm died on average at 68 ± 37.16 days (means ± standard deviation, SD), ca. 10 weeks, after entry to the group. The average age of the victims reported in the survey by the farms with gang aggression was 18.6 ± 8.2 weeks (means ± SD; range 9.5–52 weeks). The average estimated weight of the victims (from the focal farm data) was 82.9 ± 26.55 kg (means ± SD; range 31–146 kg), which would correspond to approximately 20 weeks of age. 

Three out of 23 survey respondents from farms with lethal aggression mentioned they suspected it might be related to illness of the victim (sick in general or Glässer’s disease), but there were no clinical reports to support these claims. Analysis of the body condition score (BCS) of 91 pigs from the focal farm showed that the victims were in good condition, with 73% of the pigs having a normal condition score of 3. Twelve percent of the pigs were thin (score 2) and 14% of the pigs were fat (score 4). Scores 1 (emaciated) and 5 (obese) were not recorded. The likelihood of survival after an attack was higher when the BCS of the pig was lower (χ = 6.5498, *p* = 0.04). The odds for being removed from the pen alive were 5.4 times higher for pigs with a BCS of 2 as compared to pigs with a BCS of 3 (CI: 1.412, 20.655). Pigs with a BCS of 4 did not differ from a BCS of 3 regarding the likelihood of survival (*p* = 0.81). 

In the survey, 89% of the respondents of farms with lethal gang aggression mentioned that victims had skin injuries over the whole body, whereas this was 45% for the control group. Consequently, skin lesions covering the whole body were significantly associated with the occurrence of lethal gang aggression (OR 10.2, CI: 1.548, 67.217, χ = 5.8277, *p* = 0.02). 

**Figure 3:**
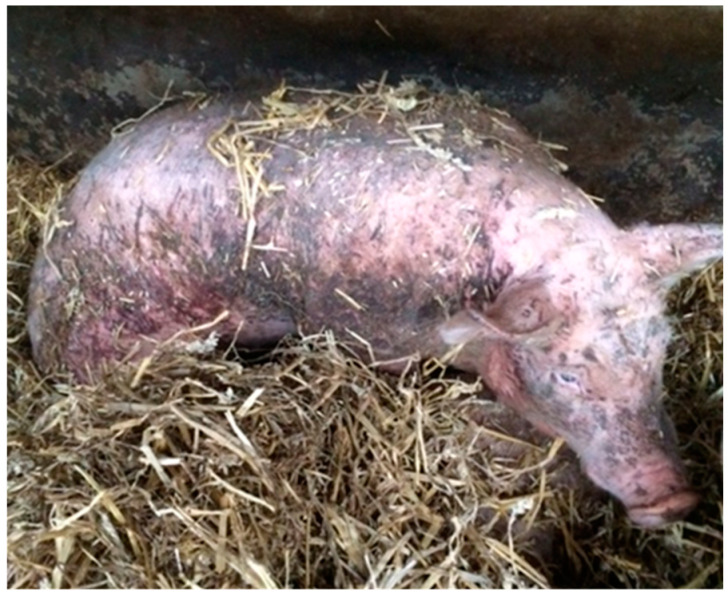
Victim found while still alive (here in recovery pen). Dark patches on the skin are, with the exception of the face, skin lesions as a result of receiving bites.

Although victims had skin lesions all over the body, the lesion scoring of the photos showed that the most severe skin injuries were predominantly on the front part of the body (face, neck, and shoulder), with the severity of lesions significantly differing by location (F_2,221_ = 18.65, *p* < 0.001). Lesions on the face (4.21 ± 0.146) were more severe than for the rear (2.41 ± 0.149), whereas lesions on the shoulder (4.43 ± 0.123) were significantly more severe than those on the middle (3.98 ± 0.118) or rear. The victims that survived the attack (*n* = 20) due to intervention by the farmer had on average a lower skin lesion score (means ± SD: 3.59 ± 0.841, range: 1.7–4.7) than those who did not survive (4.10 ± 0.836, 2.0–5.0); thus, a lower skin lesion score was associated with a higher likelihood of surviving the attack (OR 1.957, CI: 1.100, 3.482, χ = 5.2108, *p* = 0.02). For all body parts except for the rear, the survivors had a numerically lower lesion score, but on the rear they had a numerically but not significantly higher lesion score (means ± SD: 2.75 ± 1.390) than those that did not survive (2.28 ± 1.376; *p* = 0.26) (Figure 3). 

## 4. Discussion

This is the first study to report intra-group lethal gang aggression in pigs. Although 22% of the victims survived due to timely interference of the farmer, the attacks resulted in the vast majority in death. Lethal gang aggression between domestic farmed pigs was, in the current study, mostly associated with the presence of deep straw bedding and tended to occur more often in winter, but was unrelated to genetics, group size or feed type. Straw bedding, however, might be associated with differences in group size and feed type that could not be distinguished in the current sample of farm management data. Attacks occurred equally in female-only groups and mixed-sex groups, showing that this aggression is not limited to males. Literature refers to abnormal aggression when aggression exceeds a species-typical level or when the targeted area is a body part that could sustain lethal injuries, such as the head or throat [21,22]. Based on the skin lesions of victims, as well as farmers’ reports of observing this behaviour in practice (Appendix B), this form of gang aggression would classify as abnormal behaviour. 

### 4.1. Survey Sample

We acknowledge the limitation of the small sample size, where especially the control group may be less representative of the pig industry in the various countries. The variation of housing conditions reflects the UK pig industry, in line with the majority of respondents being from the UK. Given the overrepresentation of straw-based and outdoor systems, respondents may have represented a group of proactive farmers who are more concerned about animal welfare in general. Given the limitations of this first exploration of the occurrence of lethal gang aggression, we are cautious in drawing firm conclusions, especially related to straw bedding. 

### 4.2. More Aggression on Straw Bedding 

The main predictor of the occurrence of gang aggression was straw bedding. Out of 21 farms experiencing gang aggression, 13 (60%) had deep straw bedding. Straw-based systems often apply large group sizes, which may reduce aggression [23]. Although there were numerically more farms with large groups in the sample of farms with gang aggression than in the control sample, there was no influence of group size or an interaction with housing type. The absence of a relationship between straw bedding and group size might be due to the relatively small sample size in this study, and further investigation is warranted. Straw yards usually also have greater space allowances and therefore it is unlikely that crowding would have caused gang aggression [24,25]. As it is feasible that straw bedding is associated with an unknown common factor, for example a difference in management, we are cautious in assigning straw bedding as the proximate cause, given the sparse information available. However, the literature does provide a potential causality between the presence of straw bedding and heightened aggression. 

Straw is suggested to reduce aggression in pigs during regrouping, although the evidence is not conclusive [26,27]. Straw can also be a defendable resource if in scarcity [28], but the affected farms provided deep litter bedding, which means an abundance of straw was available. Straw does form a risk factor for the animals’ health due to the potential presence of mycotoxins [29,30]. Pigs are susceptible to several types of mycotoxins, especially the trichothecenes 4-deoxynivalenol (DON) and zearalenone (ZEN), which have severe consequences for pig health and immunocompetence [31,32,33]. Some mycotoxins, including those to which pigs are sensitive, can lead to gastrointestinal lesions and may negatively affect the gut microbiota [33]. In turn, the microbiota, through the microbiota–gut–brain-axis [34,35], is associated with behaviour, including elevated levels of aggression (dogs: [36]; pigs: [37]). Changes in the microbiome, which may be induced by nutritional changes, psychological stress, and other causes, can also result in changes in the animal’s olfactory signature [38] and thereby the ability of group members to recognise the individual. 

Although there is a potential relationship between straw provision and aggression, this does not fully explain the peculiar abnormal behavioural pattern seen during lethal gang aggression. Investigating the quality of the straw (and feed, as this can also contain mycotoxins and alter the microbiota) would, however, be worthwhile when lethal gang aggression occurs in order to discover the potential cause of this relationship. 

### 4.3. Seasonal Influences

On the focal farm, which reported 316 cases of lethal gang aggression, there was a tendency for more attacks in winter. This might relate to the association between deep straw bedding and gang aggression, as bedding is often increased in winter to facilitate thermoregulation. As this result stemmed from the data of only one farm, it might be that it is related to specific farm management and therefore this result is potentially not applicable across pig farms.

### 4.4. Lethal Aggression in Female-Only Groups

A unique feature of the cases reported here is that it occurred equally in female-only groups and groups of both males and females. All lethal coalitional aggression in primates has happened between males [2,5]. The current results were from pre-pubertal animals, and thus rules out that the aggression occurred due to such reproductive pressure [6,10]. In nature, pigs would live in matrilineal groups from which the males disperse. Female aggression is, in nature, mainly related to the defence of offspring. Under husbandry conditions, pre-pubertal female pigs as well as adult sows show fierce aggression in the establishment of dominance relationships [39]. Our data show that females were both victims and aggressors in a majority of cases. Attacks occurred on average at 18 weeks of age, which is around the time when gilts are at the onset of early puberty (at ca. 20 weeks of age), especially when boars are present, as this can stimulate early sexual maturity [40]. One respondent suggested a relationship with oestrus cycles in his pigs. Changes in behaviour [40] and olfactory signals [41] due to the occurrence of oestrus could potentially have occurred at the time of the attacks, but the data here show that the onset of oestrus cannot be the sole explanation as gang aggression also occurs in males. 

### 4.5. Instability in Dominance Relationships

In other species, intra-group coalitional aggression is mainly related to challenges to established dominance rank, e.g., primates: [10,11] and domestic dogs: [42]. Pigs establish dominance relationships, and commonly use fierce reciprocal fights in this process, although these are rarely fatal [19,43]. Analysis of the skin lesions of victims revealed that most injuries were on the front of the body, which is related to reciprocal aggression during non-lethal forms of aggressive interaction [44]. This is commonly an indication that the victim had actively reciprocated the aggression [45]. In this case, however, it may also be that the victim was already too exhausted to retaliate and therefore received numerous bites on its vulnerable body parts. This is a feasible explanation since the interactions involved multiple attackers. Moreover, the severity of the lesions is far beyond what would be seen in normal fights for dominance, which may be explained by the fact that multiple animals targeted one victim. The relatively fewer lesions on the rear of the body suggest that the victim was unable to escape, as lesions on the rear commonly accumulate from running away from the attacker [45]. 

In the present dataset, the skin lesions, albeit severe, did not appear as if they would be the main cause of death, and we expect that most victims died from the related stress (traumatic shock) and exhaustion, which may result in sudden cardiac failure [46] or stress-induced hyperthermia [47,48]. Victims were on average around 80 kg in body weight, which makes them more vulnerable to heat stress [49], even at moderate outdoor temperatures. Pigs with a lower body condition score (which can occur irrespective of body weight) had a higher chance of survival. It may be that these animals were discovered sooner by the farmers by chance or, for example, that the lower body condition made them more agile and capable of retreat or resilient against heat stress [49]. 

### 4.6. Lethal Aggression and Genetics

Farmers expressed concern that gang aggression may have a genetic basis. Commercial crossbred pigs may have gradually become more aggressive over time, partly due to strong genetic selection for production traits [20,50]. Our data showed that lethal gang aggression occurred in a wide variety of breeds, originating from various commercial breeding companies, as well as rare breeds that have been less subjected to genetic selection for production traits. Therefore, the genetic line and breeding company seem unrelated to the occurrence of this behaviour. This does, however, not exclude the possibility that the extreme aggression may still have a genetic base. In humans and rodents, violent aggression has been associated with specific genes and mutations, mainly related to the serotonergic and dopamine system [51,52]. Assessing gene expression and SNPs (single-nucleotide polymorphisms) in relation to lethal gang aggression, as has been done for regrouping aggression [53], would be needed to exclude the possibility that lethal aggression is related to specific genes, gene expression or mutations that can be found across breeds. 

### 4.7. Nutrition

Some farmers reported that gang aggression stopped after they changed the feed composition, with changes ranging between adding zinc oxide, magnesium, protein, or commercial feed additives with a high mineral content, of which especially those related to the amino acid tryptophan are known to reduce aggression (reviewed in [19]). Due to the lack of information on the diet formulation, we cannot exclude the possibility that the composition of diet may have an influence on gang aggression. However, we also found that lethal aggression was episodic on many farms and it is possible that management changes coincided with fluctuations in aggression caused by other factors. Due to the strong genetic selection for lean muscle growth performance, the nutritional recommendations may be inadequate for the current genetics, and the interaction between genetics and nutrition may be an underlying cause for behavioural abnormalities such as extreme aggression.

### 4.8. Welfare Implications

The occurrence of lethal gang aggression raises intriguing questions as to why members of any species would gang up to attack a known conspecific. The evolutionary benefit of lethal group aggression can be diverse [54,55]. If the victim forms a threat to the health status or fitness of the group [6], then expelling or killing the group member may in some cases outweigh the costs. For commercial pigs, the benefit is difficult to perceive since there are (presumably) sufficient resources available. However, it may be that pigs do experience a shortage of essential resources such as specific nutrients, or a temporary shortage in feed, water or space due to failure of technology or management. This may highlight welfare implications that need to be investigated at the moment of occurrence. Several farmers of affected farms reported having high certified welfare standards, including increased space allowance and outdoor access. This contradicts the idea that the behaviour would be expressed as a result of poor welfare. None of the respondents mentioned changes in the pigs’ productivity. Although the occurrences to date seem relatively sparse and infrequent, even though mortality can be high on affected farms, the welfare of the victim being bitten to death and the costs associated with the mortality require this behaviour to be taken as a serious welfare concern for the pig industry. Overall, the gang aggression reveals that there is tension in commercially housed pig populations, which is an undercurrent for behaviour that is far beyond what is natural for any species and does compel us to acknowledge that domestic pigs are more emotionally complex than commonly assumed.

## 5. Conclusions

Lethal gang aggression between pigs in commercial pig husbandry is rare but can cause high mortality rates on affected farms. The reason for its occurrence is likely multi-factorial and may relate to deep litter straw bedding or factors associated with the management of farms with this housing type. The relationship between lethal gang aggression and straw requires further study, with a larger sample size for the control group and more diverse farm types, in order to disentangle the different factors. Victims had a healthy body condition score, and the reason for their victimization remains unclear. The severity of this type of aggression, and the associated impact on animal welfare and farm economics, calls for further research. Moreover, this peculiar type of gang (and potentially coalitional) aggression gives rise to fundamental questions on evolution and animal cognition that are relevant across species.

## Figures and Tables

**Table 1:** Lesion score method, adapted for the severity of lethal gang aggression.

Score	Description
1	0–10 lesions, mostly unaffected skin
2	Approximately 1/3 of the body area is covered with lesions
3	Approximately 1/2 of the body area is covered with lesions
4	At least ¾ of the body area is covered with lesions
5	At least ½ of the body area is covered with lesions including patches of uncountable or deep lesions

**Table 2:** Farm characteristics in percentages, according to survey data from farms with lethal gang aggression (*n* = 23) and without (*n* = 19). The first row for each characteristic shows the number of responses per category. Percentages may slightly deviate from a sum of 100% due to rounding of decimals.

Farm Characteristic	with Gang Aggression	without Gang Aggression	*p*-Value
Housing conditions	*n* = 22	*n* = 17	0.01
Indoor barren	27.3	76.5	
Deep litter straw	59.1	5.9	
Outdoor/outdoor access	13.6	17.7	
Group size	*n* = 23	*n* = 17	0.80
Small groups (≤30)	34.8	70.6	
Medium groups (31–100)	30.4	29.4	
Large groups (>100)	17.4	0	
Both small and large groups	17.4	0	
Group composition	*n* = 23	*n* = 18	0.17
Females only	43.5	16.7	
Males only	4.4	0	
Mixed groups	52.2	83.3	
Genetics	*n* = 20	*n* = 13	0.42
Purebred	20.0	7.7	
Cross bred	60.0	53.9	
Duroc cross bred	20.0	38.5	
Breeding company	*n* = 22	*n* = 16	0.54
A	22.7	12.5	
B	27.3	12.5	
C	9.1	12.5	
D	27.3	0	
E	4.6	12.5	
F	0	12.5	
G	9.1	36.5	
Feed type	*n* = 19	*n* = 14	0.85
Dry feed	73.7	85.7	
Wet (liquid) feed	21.1	14.3	
Both dry and wet feed	5.3	0	
Milling own feed	*n* = 9	*n* = 9	0.35
Yes	55.6	33	

## Appendix A

**Table A1:** Questions asked in the survey.

Question	Type
**Aggression in stable (not recently mixed) groups of pigs** There has been an increase in reports about aggression in stable groups of pigs, whereby the group can turn towards one individual. The Animal Behaviour & Welfare team at SRUC (a UK-based research institute) is looking at the possible causes of this type of aggression. We would be very grateful if you could help us with the following information. Your answers will be anonymous and used for research purposes only. Many thanks for your input.	Introduction
1. Please describe the situation at your farm regarding aggression between pigs in stable groups	Open
2. How often does severe aggression in stable groups (not recently mixed) occur? Occasionally, a few times a year; About 1–2× a month; Several times a month; In phases	Single choice
3. What is the average age of the pigs in which this type of aggression occurs?	Open
4. What are the results to the victim? Severe skin lesions on whole of body; Severe skin lesions on the front mainly; Severe skin lesions on the rear mainly; Lameness; Death	Multiple choice
5. What type of breed/genetic line was used at the time when this aggression occurred? (optional to specify genetics company)	Open
6. What is the average group size in which the aggression occurs?	Open
7. What is the housing type for the affected pigs? Indoor, deep litter straw; Indoor with minimum bedding or without bedding; Outdoor; Outdoor access	Single choice
8. What is the group composition? Only boars; Only gilts; Boars and gilts; Rearing gilts	Multiple choice
9. Please describe the feeding strategy (feeder type; solid/liquid feed; feed company; mineral supplementation etc.)	Open
10. Please provide any additional information that you deem relevant.	Open

## Appendix B

**Table A2:** Quotes from the respondents in the open questions about lethal gang aggression. The underlining is annotated by the authors.

Respondent	Quote
A (2)	“Without any recognizable signs or reasons all of the sudden all pigs of a group start to bit one specific member of the group to death…Within the last 7 years I had not a single case of tail biting or any other signs of abnormal behaviour.”
	“It takes not even several hours before a victim is dead. In most cases it takes about ½ hour but not more than one hour.”
B (3)	“Target pig exhibits high pitched squeal when attacked. If a pig is not removed immediately, result is death within 24 h. After segregation/death, pen behaviour returns to normal.”
	“We have the same pigs in our conventional system (fully indoor, concrete slats) and they do not have this issue.”
	“I observed one of the targeted pigs stand up during a period of respite and immediately 2 of the bully pigs ran into the target at full speed to knock it back down to the floor.”
	“We have found that the best way to fight the problem is to have growers immediately segregate any pigs that are vocalizing a high pitched/painful sounding squeal.”
C (4)	“You could watch them and all of a sudden they would pick on one pig and they would set about it and within a few minutes it would be dead if we didn’t get to it first.”
D (25)	“…[We have] no tail biting, only what I call savaging, large group 200 to 300 head in shed with outside feeding[,] floor all concrete…usually compact fat barrow most likely to be victim, but not always.”
E (28)	“The group will target one pig and kill it.”
F (32)	“They choose one pig and bully it to death. Later on sometimes they choose a next animal to bully.”
